# Dental pain perception and emotional changes: on the relationship between dental anxiety and olfaction

**DOI:** 10.1186/s12903-023-02864-9

**Published:** 2023-03-26

**Authors:** Sarah Abdulaziz Mohammed Alkanan, Hadeel Saleh Alhaweri, Ghada Amin Khalifa, Shaimaa Mohamed Saeed Ata

**Affiliations:** 1 Resident Dentist, Qusaiba Hospital, Qassim, Saudi Arabia; 2 Resident Dentist, Aljreir Alshamaly Primary Health Care Center, Qassim, Saudi Arabia; 3 Professor of Maxillofacial Surgery and Diagnostic Science, College of Dentistry, Qassim University, Qassim, Saudi Arabia; 4 Lecturer of Community Dentistry and Oral Epidemiology, College of Dentistry, Qassim University, Qassim, Saudi Arabia

**Keywords:** Dental pain, Dental anxiety, Aroma therapy, Lavender, Essential oil

## Abstract

**Background:**

The purpose of the study was to determine to what extent olfactory aromatherapy reduces the intensity of dental pain and the level of dental anxiety. It also attempted to corelate between olfactory aromatherapy, stages of dental visits, and various dental procedures.

**Methods:**

Female patients were enrolled in a randomized controlled study. Olfactory aromatherapy was performed using lavender oils. Patients were randomly assigned to one of two groups: the lavender group, in which patients inhaled 2% lavender vapors, and the control group, in which patients inhaled water vapors. Pain score, anxiety score, and changes in vital signs were among the predictable variables. Anxiety and pain were assessed using the Modified Dental Anxiety Scale (MDAS), Speilberger State-Trait Anxiety Inventory (STAI), and visual analog scales (VAS). The vital signs were systolic (SBP) and diastolic (DBP), heart rate (HR), respiratory rate (RR), and oxygen saturation (Spo2). Variables were evaluated before inhalations, 20 min after inhalations, at the end of settings, and on the following day.

**Results:**

Each group had 175 participants. Pain and anxiety scores were significantly reduced, and all vital signs improved, except for DBP. The MDAS, STAI, and pain scores are reduced by 3.4, 4.2, and 2.4 times, respectively, compared to the control group. Olfactory aromatherapy had the greatest impact during the phase of waiting rooms.

**Conclusion:**

When compared to the control group, olfactory aromatherapy reduces anxiety scores three to four times more. Pain perception is reduced by twice as much as in the control group. It also significantly reduces the anxiety associated with minor to moderately stressful dental procedures.

**Supplementary Information:**

The online version contains supplementary material available at 10.1186/s12903-023-02864-9.

## Introduction

Dental anxiety and fear are defined as an abnormal fear of going to the dentist [1, 2]. Dental anxiety is the fifth most common type of anxiety [3, 4]. It is the primary impediment to receiving proper dental treatment. Anxiety is triggered in dental clinics by a variety of factors, including sights (needles and blood), sounds of hand pieces (preparations), smells (cut dentin and bone, materials such as eugenol), and sensation (high-recurrence vibration), all of which stimulate the sensory system in the brain [5, 6]. To control and minimize pain perception and associated emotional changes, various techniques are used to manage dental fear and anxiety. Psychotherapeutic and/or pharmacological interventions are included. Anxiolytics are dangerous, necessitate specialized equipment, and may be contraindicated in certain patients [7, 8].

Aromatherapy is the use of essential oils for therapeutic purposes, and it has received attention as an alternative medicine method to control anxiety during medical settings [9]. It entails inhaling, absorbing, or ingesting essential oils to alleviate anxiety [10]. The sense of smell can induce chemical changes in the human body that range from pleasant to repulsive. So, the smell is used to improve health; this is known as olfactory aromatherapy, which is a type of complementary medicine that uses smell. There have been few studies that investigate the relationship between olfactory aromatherapy, pain perception, and anxiety in dental settings.

To the best of our knowledge, the majority of those articles only stated whether or not olfactory aromatherapy is effective, but they made no predictions about its potency. Furthermore, the majority of those studies searched the impact of olfactory aromatherapy on local anesthesia and tooth extraction procedures, but not on other dental procedures. Moreover, they did not investigate at which stage of dental visits olfactory aromatherapy is most effective. As a result, this study was designed to answer the following questions: 1) What is the potency of olfactory aromatherapy in reducing pain perception and anxiety during dental visits? 2) Which dental procedure is significantly influenced by the use of olfactory aromatherapy? And 3) When is the greatest effect of olfactory aromatherapy recorded in dental settings? Therefore, this study amid to investigate to what extent olfactory aromatherapy is able to reduce the intensity of dental pain and the level of dental anxiety. It also attempted to corelate between olfactory aromatherapy, stages of dental visits, and various dental procedures.

## Materials and methods

### Study design

A prospective randomized double-blind controlled parallel grouped clinical trial was conducted on dental patients who attended the outpatient Dental clinics at Qassim University's College of Dentistry between March 2018 and January 2021. For olfactory aromatherapy, lavender essential oil was chosen to stimulate smell. The patients were randomly assigned to one of two groups: the control group, in which the patients inhaled plain distal water vapors, and the lavender group, in which patients inhaled lavender oil vapors. Age, gender, and time were avoided as confounding factors because they have a large impact on pain perception and anxiety as reported by many authors [11–13]. As a result, patients were chosen based on the inclusion and exclusion criteria listed in Table 1. The required permissions and informed consent were obtained; the study was approved by the Dental Ethical Committee, College of Dentistry, Qassim University, Saudi Arabia on 20/3/2018 with approval registry number "EA/3006/2018." On 11/5/2022, the study was retrospectively registered at clinicalTrials.gov, with the identifier "NCT05369936."

**Table 1:** The study’s inclusion and exclusion criteria

Inclusion Criteria	Exclusion Criteria
Adult females over 18 years	Males
females who attended morning dental clinics at 9 am	Females who had: - Allergies - Bronchial asthma - Common cold - Pulmonary diseases - Migraine
Females who were liable for: **-** Administration of the local anesthesia **-** Teeth preparation either for filling procedures or crown preparations **-** Endodontic treatment **-** Teeth extraction **-** Subgingival curettage and scaling	Females who taking: - Antidepressants - Anxiolytic drugs - Opioids - Other medications that affect emotional responses
	Pregnant female

### Sampling, randomization, and blindness

The sample size was calculated using the PASS 16 Power Analysis and Sample Size Software version 2018 (NCSS, LLC. Kaysville, Utah, USA, ncss.com/software/pass). The results indicated that the total sample size for the study has to be 338 subjects (169 patients in each group), with = 0.05 and power = 80%. To account for any dropouts, the sample size in each group was increased to 175 participants. The patient sequences were used for randomization. Patients with even numbers were assigned to the lavender group, while those with odd numbers were assigned to the control group. To ensure the validity of the study's findings, both groups were treated on different days to guarantee that the smell of lavender oil was completely removed from the clinics and waiting room. The following steps were taken to achieve blindness: 1) The patients, dentists who treated them, and evaluators who interviewed them to measure the study's variables were not informed about the study, so the vaporizers were placed as part of the clinic's and waiting room's equipment throughout the study's period, and 2) The investigators who reviewed the questionnaires and the statistician who analyzed the study's results only knew the study's groups as A and B.

### Study variables

The predictable variable was the ability of olfactory aromatherapy to reduce dental pain and anxiety. This ability was calculated in terms of pain perception and emotional changes during phases of dental settings where different dental procedures were performed. The intensity of pain felt before, during, and after dental procedure was used to evaluate pain perception. To determine emotional changes, the anxiety level and the measurements of patients’ vital signs were measured before, during, and after dental procedures as well. The primary outcome variables were the changes in the preoperative dental anxiety, dental pain intensity, and baseline measurements of the patients’ vital signs (SBP, DBP, HR, RR, Spo2). The secondary outcome variables included the phase of the dental setting and dental procedures where olfactory aromatherapy had the greatest effect. Furthermore, the need for postoperative analgesics or anxiolytics after dental settings was documented as an additional record.

### Data collection

The patients’ demographic information was recorded. The MDAS questionnaire (a simplified scale with five questions and scores ranging from one to five) and the STAI questionnaire (a scale consists of 40 questions and has a score range of 20 to 80) were used to assess the level of dental anxiety. The intensity of pain was measured using VAS, with scores ranging from 0 to 100 mm. All of the aforementioned scales were compiled into a single questionnaire, which was given to each patient four times. The first questionnaire was given to ever patient an hour before exposure to vapors in the waiting room (Phase I), and its results were used as baseline measurements. The second one was obtained 20 min after vapor exposure in setting rooms (Phase II).

Following the completion of the dental procedures, the third questionnaire was completed (Phase III). The fourth questionary was obtained on the following day after dental procedures (Phase IV). Phases I and II were completed in the waiting room, while phases III and IV were completed in dental clinics. Two senior staff members were in charge of collecting and reviewing the questionnaires, and each was assigned to one of the study's groups. The same healthcare staff measured the patients' vital signs four times in the same order as the questionnaires. The SBP and DBP were measured in millimeters of mercury using a sphygmomanometer and stethoscope. The patients were asked to stand up straight, and the sphygmomanometer cuff was inserted around their upper left arms, with a 2.5-inch distance between the antecubital fossa of the arm and the lower edge of the cuff. The blood pressure was measured after the stethoscope was placed over the brachial artery. The HR, RR, and Spo_2_ were measured using a finger pulse oximeter.

### Intervention

After completing the first questionnaire and measuring the vital signs (phase I), 20 drops (0.25 mL) of 100% organic essential lavender oil (Lavandula angustifolia, 10 mL, Australian Certified Organic Pty Ltd., Brisbane, Australia) and 50 mL of distal water were added to an electrical aromatherapy vaporizer near the patient's chair at a distance of 20 cm in the waiting room. After 20 min, the second questionnaire was given the patients and their vital signs were measured (phase II). Then, the patient was transferred to dental clinics, where the lavender vaporizer was placed 20 cm away from the dental unit. Every 20 min, 20 drops of lavender oil were added to the vaporizer until the dental treatment was completed. Before the patient left the clinic, the third questionnaire was completed and measurements of the vital signs were also recorded (phase III). For phase IV of the study, all patients were given 0.25 mL of lavender oil from the same bottle to use at home. Three drops of lavender oil were deposited on a cotton pad, and the patients were instructed to inhale it for five minutes from a distance of ten centimeters three times per day until they returned the following day to complete the fourth questionnaire.

On the day of the control group, the waiting room and clinics were thoroughly ventilated to ensure that the lavender scent was completely removed from the environment. The vaporizer was also placed 20 cm away from the patient's chair in the waiting room, but it was only filled with plain distal water. All of the above-mentioned lavender group protocol was also applied to the patients in the control group, except for using olfactory aromatherapy at home. They were only instructed to perform the standard postoperative dental care at home. All dental procedures, in both groups, were performed under local anesthesia with vasoconstrictor using nerve block local anesthetic techniques (Mepivacaine 2% and epinephrine 1:100,000). All patients were instructed to adminstrate diclofenac sodium 50 mg twice daily if they experienced postoperative pain.

### Statistical analysis

Our data was analyzed using SPSS Version 25.0 for Windows (SPSS Inc., Chicago, IL, USA). All participants' baseline characteristics were expressed using descriptive statistics. The variables were summarized into mean and standard deviation, and the results were analyzed using the two-sample t and ANOVA tests. The two-sample *t*-test was used to compare baseline differences between groups. The paired *t*-test was used to compare variables before and after intervention. *P*-values less than 0.05 and confidence intervals of 95% were considered significant.

## Results

### Demographic data and baseline assessment

**Fig. 1:**
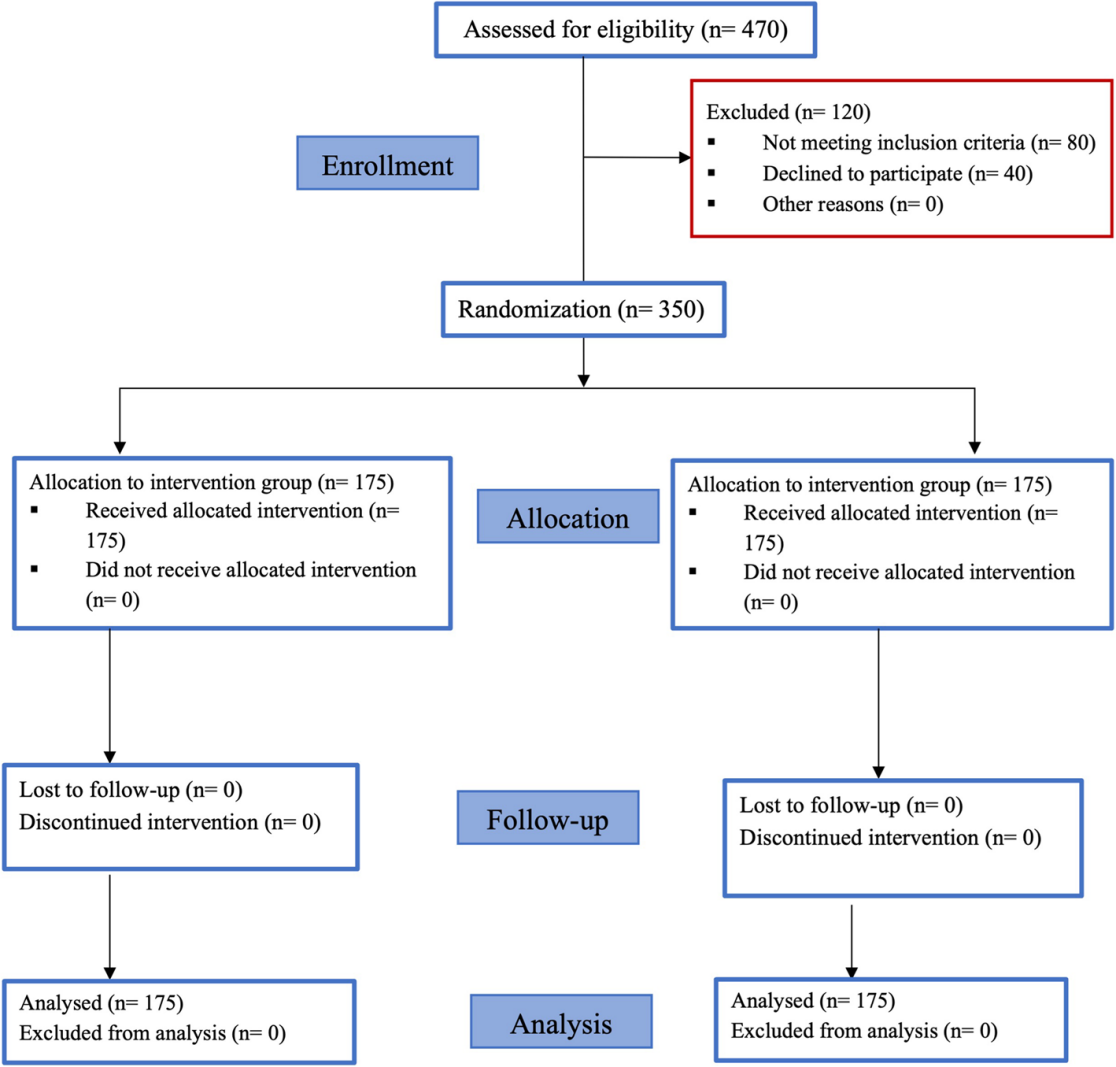
The study’s flow diagram demonstrating dental patients who participated in each group

Only 350 female subjects out of 470 met the inclusion criteria for the study. There were 175 patients in each group, with an average age of 23.5 ± 1.12 years. None of the patients were dropped off and all the study’s phases were completed (Fig. 1). Table 2 summarizes the study's demographic data and baseline parameters of the patients. In terms of all preoperative data, both groups were homogeneous. There were no adverse reactions such as nausea, severe hypotension, or syncope observed due to inhalation of lavender oil. Each questionnaire needed patients seven to ten minutes to be answered, and the investigators scored it in five minutes. The first phase's results (prior to inhaling vapors) revealed that all anxiety levels ranged from moderate to extremely high. This was reflected in the intensity of the pain, which ranged from mild to severe. The baseline vital sign measurements showed a slight increase, except for the Spo_2_ percentage, which showed a slight decrease in blood oxygen saturation.

**Table 2:** The patients’ demographic data and results of the baseline of the study’s variables (phase I)

Patients (*n* = 350)	Lavender Group (*n* = 175)	Control Group (*n* = 175)	*P*- value
Age (in years):			
Mean ± SD	23.98 ± 0.109	23.72 ± 0.201	0.220
Standard Error	0.31	0.30	
Confidence level (95%)	23.98 ± 0.606	23.72 ± 0.595	
Gender	Female	Female	-
Nationality:			
-Saudi	120 (34.29%)	115 (32.86%)	0.123
-None- Saudi	55 (15.71%)	60 (17.14%)	
Educational level:			
-Elementary school	10 (2.86%)	8 (2.29%)	0.211
-Middle school	24 (6.86%)	22 (6.28%)	
-High school	38 (10.86%)	40 (11.43%)	
-College	101(28.85%)	102 (29.14%)	
-Post graduate	2 (0.57%)	3 (0.86%)	
Social status:			
-Single	123 (35.15%)	129 (36.86%)	0.142
-Married	34 (9.71%)	31 (8.86%)	
-Divorced	14 (4%)	12 (3.43%)	
-Widow	4 (1.14%)	3 (0.85%)	
Dental Procedures:			
-Local Anesthesia	175 (50%)	175 (50%)	0.203
-Crown preparations	50 (14.29%)	48 (13.71%)	
-Subgingival scaling	44 (12.57%)	50 (14.29%)	
-Cavity preparations	26 (7.43%)	30 (8.57%)	
-Pulp Extirpation	27 (7.71%)	23 (6.57%)	
-Extractions	28 (8%)	24 (6.86%)	
Baseline SBP (mmHg)			
(Mean ± SD)	140.56 ± 16.53	142.32 ± 12.36	0.763
Baseline DBP (mmHg)			
(Mean ± SD)	81.63 ± 11.24	80.45 ± 10.23	0.320
Baseline HR (beat/min)			
(Mean ± SD)	76.61 ± 10.21	76.81 ± 11.23	0.654
Baseline RR (breathes/min)			
(Mean ± SD)	17.82 ± 1.30	17.80 ± 1.34	0.764
Spo_2_ (%)			
(Mean ± SD)	96.65% ± 1.78	96.22% ± 2.89	0.879
Baseline MDAS			
Score (Mean ± SD)	7.43 ± 0.121	7.73 ± 0.152	0.122
Level:			
-Fairly anxious:	89 (25.43%)	87 (24.86%)	0.432
-Very anxious:	50 (14.29%)	54 (15.43%)	
-Extremely anxious	36 (10.29%)	34 (9.71%)	
Baseline STAI			
Score (Mean ± SD)	75.54 ± 10.65	76.58 ± 11.12	0.111
Degree:			
- Moderate	89 (25.43%)	96 (27.43%)	0.213
- High	86 (24.57%)	79 (22.57%)	
Baseline Pain Score			
Score (Mean ± SD)	89.78 ± 1.23	93.21 ± 2.23	0.233
Intensity:			
-Mild pain	75 (21.43%)	69 (19.71%)	0.212
-Moderate pain	62 (17.71%)	68 (19.43%)	
-Severe pain	38 (10.86%)	38 (10.86%)	

*SD* Standard Deviation, *SBP* Systolic Blood Pressure, *DBP* Diastolic Blood Pressure, *HR* Heart Rate, *RR* Respiratory Rate, *Spo2* Oxygen Saturation, *MDAS* Modified Dental Anxiety Scale, *STAI* Speilberger State-Trait Anxiety Inventory, *VAS* Visual Analog Scale

### Anxiety and pain scores assessment in waiting room

The baseline scores of the STAI, MDAS, and pain, in the lavender group, were significantly lower in the second phase of the study (after inhaling vapors for 20 min) (Table 3). The mean change in the STAI was—4.7 ± 8.60, while the MDAS and pain scores were -2.21 ± 0.13 and—8.82 ± 3.34, respectively. According to the ANOVA test results, lavender aromatherapy significantly reduced the MDAS, STAI, and pain scores by 3.4, 4.2, and 2.4 points, respectively, compared to the control group (Table 4). Furthermore, all vital signs were also significantly improved, except for the DBP. In the control group, all of the examined parameters were improved, but without any statistical significance, except for the HR.

**Table 3:** Comparison of the study’ variables at the baseline (phase I) and the end of the second phase in the lavender and control groups

Parameters	Lavender Group (Mean ± SD)				Control Group (Mean ± SD)			
	Phase I	Phase II	Change	*P*- value	Baseline	Phase 2	Change	*P*-value
SBP	140.56 ± 16.53	133.16 ± 12.20	- 7.4 ± 12.34	0.021	142.32 ± 12.36	137.09 ± 10.04	- 5.23 ± 11.24	0.143
DBP	81.63 ± 11.24	80.43 ± 6.87	- 1.2 ± 10.25	0.062^*^	80.45 ± 10.23	79.69 ± 9.98	- 0.76 ± 10.54	0.239
HR	76.61 ± 10.21	69.26 ± 7.76	- 7.35 ± 9.21	0.041	76.81 ± 11.23	71.68 ± 5.67	- 5.13 ± 2.98	**0.042**^**$**^
RR	17.82 ± 1.30	16.48 ± 0.78	- 1.34 ± 0.12	0.041	17.80 ± 1.34	16.82 ± 6.98	- 0.98 ± 2.14	0.214
Spo_2_	96.65% ± 1.78	97.98% ± 1.96	+ 1.33% ± 0.25	0.032	96.22% ± 2.89	96.78% ± 0.87	+ 0.56% ± 0.29	0.367
MDAS	7.43 ± 0.121	5.22 ± 0.54	- 2.21 ± 0.13	0.012	7.73 ± 0.15	7.30 ± 0.98	- 0.43 ± 0.21	0.340
STAI	75.54 ± 10.65	70.84 ± 9.91	- 4.7 ± 8.60	0.011	76.58 ± 11.12	76.02 ± 0.87	- 0. 56 ± 0.43	0.653
Pain score	89.78 ± 1.23	80.96 ± 0.87	- 8.82 ± 3.34	0.023	93.21 ± 2.23	90.84 ± 4.76	- 2.37 ± 0.67	0.087

*SD* Standard Deviation, *SBP* Systolic Blood Pressure, *DBP* Diastolic Blood Pressure, *HR* Heart Rate, *RR* Respiratory Rate, *Spo*_*2*_ Oxygen saturation, *MDAS* Modified Dental Anxiety Scale, *STAI* Speilberger State-Trait Anxiety Inventory, *VAS* Visual Analog Scale

^*^Not Significant

^$^Significant

**Table 4:** The amount of change in vital signs, anxiety, and pain scores between the first and second phases

Parameters	Lavender Group (Mean ± SD)	Control Group(Mean ± SD)	*P*- Value	ANOVA test		
				Coefficient	95% CI	*P*-value
SBP changes	- 7.4 ± 12.34	-5.23 ± 11.24	0.124	- 2.9	-8.5, 2.8	0.34
DBP changes	- 1.2 ± 10.25	-0.76 ± 10.54	0.231	- 1.5	-3.5, 5.6	0.62
HR changes	- 7.35 ± 9.21	-5.13 ± 2.98	0.063	- 2.4	- 5.7, 1.8	0.27
RR changes	- 1.34 ± 0.12	-0.98 ± 2.14	0.240	- 0.9	- 1.7,0.03	0.35
Spo_2_	+ 1.33% ± 0.25	+ 0.56% ± 0.29	0.154	1.1	1.3, 0.21	0.21
MDAS changes	- 2.21 ± 0.13	-0.43 ± 0.21	0.021^*^	**- 3.4**^**^**^	-5.7,—0.8	0.014^*^
STAI changes	- 4.7 ± 8.60	-0. 56 ± 0.43	0.023^*^	**- 4.2**^**^**^	- 6.9,—0.9	0.012^*^
Pain score changes	- 8.82 ± 3.34	- 2.37 ± 0.67	0.012^*^	**- 2.4**^**^**^	- 4.7,—0.5	0.023^*^

*SD* Standard Deviation, *SBP* Systolic Blood Pressure, *DBP* Diastolic Blood Pressure, *HR* Heart Rate, *RR* Respiratory Rate, *Spo*_*2*_ Oxygen saturation, *MDAS* Modified Dental Anxiety Scale, *STAI* Speilberger State-Trait Anxiety Inventory, *VAS* Visual Analog Scale

^*^ Significant ANCOVA, analysis of covariance; *95% CI* 95% confidence interval

^^^ The pleasant scent of the lavender significantly reduced the MDAS, STAI, and pain scores 3.4, 4.2, and 2.4 more than the control group, respectively

### Changes in anxiety and pain scores at the end of dental procedures

Table 5 compares the second and third phases of the research. The lavender aromatherapy improved all parameters better than the distal water, but the differences were only significant for anxiety, pain intensity, and SBP. At the end of the dental procedures, lavender aromatherapy reduced scores by 1.2 (for MDAS), 2.2 (for STAI), and 1.4 (for pain intensity) times more than the control group (Table 6). Those findings revealed that the olfactory aromatherapy had the greatest effect in the waiting room.

**Table 5:** Comparison of the study’ variables between the second and third phases of the study within each group

Parameters	Lavender Group (Mean ± SD)				Control Group (Mean ± SD)			
	Phase II	Phase III	Change	P- value	Phase 2	Phase 3	Change	*P*-value
SBP	133.16 ± 12.20	130.34 ± 1.23	-2.82 ± 0.03	0.031^*^	137.09 ± 10.04	135.85 ± 1.34	- 1.24 ± 1.29	0.153
DBP	78.43 ± 6.87	77.98 ± 0.98	- 0.55 ± 0.25	0.132	77.69 ± 9.98	77.25 ± 0.23	- 0.44 ± 0.94	0.165
HR	69.26 ± 7.76	68.84 ± 0.43	- 0.42 ± 0.81	0.061	71.68 ± 5.67	70.68 ± 0.23	- 1.00 ± 0.58	0.252
RR	16.48 ± 0.78	16.12 ± 0.96	- 0.36 ± 0.12	0.101	16.82 ± 6.98	16.21 ± 0.36	- 0.61 ± 0.14	0.143
Spo_2_	97.98% ± 1.96	98.23% ± 0.34	+ 0.25% ± 0.25	0.112	96.78% ± 0.87	96.99% ± 0.75	+ 0.21% ± 0.26	0.367
MDAS	5.22 ± 0.54	3.47 ± 0.84	- 1.75 ± 0.13	0.012^*^	7.30 ± 0.98	6.96 ± 0.57	- 0.34 ± 0.21	0.140
STAI	70.84 ± 9.91	66.41 ± 0.35	- 4.43 ± 8.60	0.001^*^	76.02 ± 0.87	75.45 ± 0.85	- 0.57 ± 0.63	0.136
Pain score	80.96 ± 0.87	69.31 ± 0.42	- 11.56 ± 0.25	0.003^*^	90.84 ± 4.76	89.14 ± 0.64	- 1.7 ± 0.37	0.077

*SD* Standard Deviation, *SBP* Systolic Blood Pressure, *DBP* Diastolic Blood Pressure, *HR* Heart Rate, *RR* Respiratory Rate, *Spo*_*2*_ Oxygen saturation, *MDAS* Modified Dental Anxiety Scale, *STAI* Speilberger State-Trait Anxiety Inventory, *VAS* Visual Analog Scale

^*^Significant

**Table 6:** Percentage of changes in the vital signs, anxiety and pain scores of the study’s groups between second and third phases

Parameters	Lavender Group (Mean ± SD)	Control Group (Mean ± SD)	*P*- Value	ANOVA test		
				Coefficient	95% CI	*P*-value
SBP changes	-2.82 ± 0.03	- 1.24 ± 1.29	.241	- 2.9	-6.5, 1.8	.323
DBP changes	- 0.55 ± 0.25	- 0.44 ± 0.94	.131	- 1.5	-1.4, 6.6	.23
HR changes	- 0.42 ± 0.81	- 1.00 ± 0.58	.073	- 2.4	- 3.4, 1.4	.17
RR changes	- 0.36 ± 0.12	- 0.61 ± 0.14	.140	- 0.9	- 1.5,0.08	.29
Spo_2_	+ 0.25% ± 0.25	+ 0.21% ± 0.26	.104	1.1	1.3, 0.21	.12
MDAS changes	- 1.75 ± 0.13	- 0.34 ± 0.21	.031^*^	**- 1.2**^**^**^	-2.4,—0.4	.054^*^
STAI changes	- 4.43 ± 8.60	- 0.57 ± 0.63	0.013^*^	**- 2.2**^**^**^	- 5.8,—0.4	.022^*^
Pain score changes	- 11.56 ± 0.25	- 1.7 ± 0.37	0.012^*^	**- 1.4**^**^**^	- 2.7,—0.6	.024^*^

*SD* Standard Deviation, *SBP* Systolic Blood Pressure, *DBP* Diastolic Blood Pressure, *HR* Heart Rate, *RR* Respiratory Rate, *Spo*_*2*_ Oxygen saturation, *MDAS* Modified Dental Anxiety Scale, *STAI* Speilberger State-Trait Anxiety Inventory, *VAS* Visual Analog Scale

^*^Significant *ANCOVA* analysis of covariance, *95% CI* 95% confidence interval

^^^The pleasant scent of the lavender significantly reduced the MDAS, STAI, and pain scores 1.2, 2.2, and 1.4 more than the control group, respectively

### Correlation between dental procedures and olfaction

Olfactory aromatherapy had a significant effect on pain perception and emotional changes associated with all dental procedures except cavity preparations, where the difference was insignificant (*p*-value = 0.10). Despite this, the *p*-value for pain intensity was 0.05, indicating marginal significance (Table 7). Finally, olfactory aromatherapy significantly reduced postoperative pain (*p*-value = 0.02). Only 19 patients in the lavender group needed postoperative analgesics, compared to 48 in the control group.

**Table 7:** The difference between the study’s groups in regard to the dental procedures and pain scores during dental settings (Phase IV)

Procedure	Lavender Group	Control Group	*P*-value
Local Anesthesia:			
-Pain score (Mean ± SD)	33.45 ± 2.23	47.32 ± 1.25	0.041
-Anxiety level:			
Before	Moderate anxiety	Moderate anxiety	0.023
After	Mild anxiety	Moderate anxiety	
Crown preparations:			
-Pain score (Mean ± SD)	29.87 ± 2.15	45.32 ± 2.35	0.032
-Anxiety level:			
Before	Mild anxiety	Mild anxiety	0.042
After	Low anxiety	Mild anxiety	
Subgingival scaling			
-Pain score (Mean ± SD)	21.54 ± 0.24	27.32 ± 0.28	0.001
-Anxiety level:			
Before	Low Anxiety	Low anxiety	0.043
After	No	Low anxiety	
Cavity preparations			
-Pain score (Mean ± SD)	5.24 ± 0.46	10.55 ± 0.32	0.050
-Anxiety level:			
Before	Low anxiety	Low anxiety	0.102^*^
After	No	No	
Pulp extirpation			
-Pain score (Mean ± SD)	4.36 ± 0.47	7.32 ± 0.42	0.023
-Anxiety level:			
Before	Mild anxiety	Mild anxiety	0.022
After	No anxiety	Low anxiety	
Extractions			
-Pain score (Mean ± SD)	21.46 ± 1.23	45.49 ± 0.23	0.021
-Anxiety level:			
Before	Moderate anxiety	Moderate anxiety	0.001
After	Mild anxiety	Moderate anxiety	

^*^: None significant

## Discussion

Anxiety develops when patients are subjected to stressful situations, such as medical interventions [14, 15]. Our findings revealed that the anxiety associated with tooth extractions or receiving local anesthesia was greater than that associated with other dental procedures. This is consistent with other studies [8, 16]. The American Psychiatric Association classified dental anxiety as a blood-injection-injury phobia, which is consistent with our findings [17, 18]. This could be due to gag reflex, choking, injection action, inability to see what is going on inside the mouth, or a strong aversion to the sight or thought of blood, as reported by others [16, 19].

In general, there is no difference between the study's findings and those of other studies [20–24]. The overall findings of the study revealed that olfactory aromatherapy significantly reduces dental anxiety and pain intensity, resulting in better control of pain perception and emotional changes associated with dental visits. This is consistent with the findings of Paradopo et al. [14]. At the end of the second phase of the study, olfactory aromatherapy reduced the MDAS and STAI by 3.4 and 4.2 times, respectively, compared to the control group. It is 2.4 times more effective than the control group in terms of pain intensity. At the end of the third phase of the study, olfaction aromatherapy reduced the MDAS, STAI, and pain intensity by 1.2, 2.2, and 1.4 times, respectively, compared to the control group. This implies that olfactory aromatherapy has a significant effect on both pain intensity and anxiety level, but it is more effective at reducing anxiety than pain intensity. Furthermore, prior to the start of dental treatment, olfactory aromatherapy has the greatest impact.

On the other hand, the study's findings contradict the results of Muzzarelli et al. [25] who stated that olfactory aromatherapy has no significant effect on anxiety levels. Furthermore, Kritsidima et al. [26] concluded that, olfactory aromatherapy only affects the state anxiety and not the cognitive aspect of anxiety. As previously stated, state anxiety is a temporary emotional condition characterized by apprehension, tension, and fear about a specific temporary situation, whereas cognitive anxiety is associated with negative thoughts about this situation. State anxiety is characterized by muscle tension, increased heart rate, sweating, and a stressful feeling. In dental clinics, controlling state anxiety is more important than controlling cognitive anxiety, because controlling state anxiety enables dentists to control patients' behaviors on the spot, allowing them to perform dental procedures more successfully. Kritsidima et al. [26] agreed with our findings, where they concluded that olfaction aromatherapy is used to reduce anxiety on the spot rather than as a treatment option.

Lavandula angustifolia (lavender essential oil), the main constituents of which are linalool and linalyl acetate, was used in our study to stimulate olfaction in patients. Linalool stimulates serotonin secretion in the brain, which acts as a sedative and improves the patient's mood. Linalyl acetate, on the other hand, is a narcotic that helps patients to control their behaviors. Both components act as anxiolytic, and thus lavender oil reduces anxiety in the patients [27, 28]. However, our explanation contradicts the findings of other authors, who stated that there is no evidence that pleasant odors have an inhibitory effect on anxiety, and the effect is still unknown [26, 29].

Understanding the physiology of emotional responses to different stimuli, as well as how the anxiety develops in humans, could aid in explaining the findings of our study. Olfaction, like any other sensory stimuli, affects the limbic system in the brain, which regulates emotional changes either positive or negative [30]. The amygdala is a part of the limbic system which is an important mediator of many aspects of emotional behaviors. It is responsible for processing positive emotions such as happiness, and negative ones such as fear and anxiety. Amygdala is activated by sensory stimulation, specifically the olfactory, auditory, and visual cortices. Anatomically, the olfactory bulb is intimately and directly connected to the amygdala. As a result, pleasant or unpleasant odors have a greater impact on human emotional responses than other sensory sensations because of its effect on the amygdala [27, 31].

If the odors are anxious, the amygdala stimulates the hypothalamus, which stimulates the pituitary gland, which stimulates the adrenal gland to secrete stress hormones (adrenaline, noradrenaline, catecholamines, and corticosteroids). Stress hormones are responsible for the fight-or-flight response, which protects the body from anxious stimuli. Cortisol also makes the patient more alert and sensitive to stimulation. If the stimulus is chronic and persistent, anxiety will develop as a result of the constant release of stress hormones, resulting in negative behavioral and emotional responses. Anxiety is also experienced as a result of sustained cortisol secretion, which inhibits serotonin secretion. [27, 32].

On the other hand, the pleasant sent activates olfactory receptor cells connected to the olfactory bulb, which then sends signals to the amygdala and olfactory cortex. These activities cause the amygdala to initiate good memories, resulting in the release of anxiolytic neurotransmitters such as endorphins and serotonin, which improve mood and produce sedation. Furthermore, the pleasant sent reduces anxiety by inhibiting stress hormone secretion [28]. Moreover, Bombail [31] reported that pleasant odors produce anxiolytic effects in the brain by altering the gamma-aminobutyric acid receptors, similar to benzodiazepines, which are anxiolytic substances. Additionally, other authors claim that pleasant odors cause relaxation by modulating the adenosine monophosphate cycle, which is responsible for sedation and relaxation [33]. All of the aforementioned methods indicate that olfaction has a significant impact on the anxiety production system in the brain via a variety of mechanisms.

In terms of pain perception, the study's findings revealed that olfaction aromatherapy significantly reduces the intensity of pain and eliminates the need for postoperative analgesics, as also reported by Moss et al. [34]. This could be due to the ability of the pleasant sent to regulate the cognition, mood, and behavior of patients. Controlling those emotional changes and anxiety raises the pain threshold, which reduces pain perception. Many authors have shown that mental stress affects the physiological frameworks of the body by increasing cortisol production in the blood. Cortisol also makes patients more aware of their surroundings, which increases pain perception in the brain [20, 31, 35]. As a result, we believe that reducing anxiety indirectly decreases pain perception. According to the American Psychological Association, the nervous system is intimately connected to the stress-anxiety system in the body. Stress and anxiety, as a result, influence pain perception and cause hyperalgesia, which exaggerates pain. In contrast, other authors stated that the effect of pleasant odor is limited to anxiety levels and has no effect on pain perception. This is because it only provides relaxation and not analgesia [36].

Regarding hemodynamic changes, the inhalation of the olfactory aromatherapy was significantly improving all the vital signs, except DBP. The same finding was also reported by Stanley et al., [15]. On the other hand, other authors found that its effect is only limited to SBP [37]. This controversy could be due to the difference between studies regarding the type of the inhaled oil, its concentration, and inhalation period. This authors’ explanation is in agreement with the opinion of Ebrahimi et al., [20].

The clinical importance of our study is that, up to our knowledge, it is the first study that predict to what extent the olfactory aromatherapy affects anxiety and pain perception during dental settings. Furthermore, because of the study's design and intervention, the findings could be generalized. Moreover, the findings of this study may encourage dentists to consider using lavender oil aromatherapy as a simple complementary therapy in dental settings to reduce dental anxiety and pain and avoid the risks of anxiolytic drugs. Those drugs have drawbacks, such as abuse/dependence liability, amnesia, clinical effect delay, sedation, and dizziness, all of which affect clinical adherence. Also, olfactory aromatherapy is a simple, inexpensive, non-invasive technique with few side effects, can be used to manage anxiety and pain in medical settings.

The study has some limitations, such as a small sample size, and the patients were treated by a variety of dentists who took different approaches to their patients' behavior and attitudes. Furthermore, the results of the study were obtained via a questionnaire, which had drawbacks. It has been demonstrated that the patient's mode influences his or her responses to questions. Despite this, our study has many strengths, such as the absence of confounders such as age, gender, and time lapse. Its preferred random control trial design, as well as the fact that it is a single institutional study.

## Conclusion

Our findings suggest that olfactory enrichment and conditioning through a pleasant scent could be used to provide positive emotional responses and reduce pain perception in dental patients. After inhaling the pleasant scent, it can reduce dental anxiety by three to four times and pain by two times more than the control group. It also significantly reduces fear of dental procedures with moderate pain and anxiety scores, such as tooth extractions and the administration of local anesthesia. As a result, it can be used in conjunction with analgesics and anxiolytics to alleviate pain perception and dental anxiety.

## Supplementary Information

**Additional file 1.** Questionnaire.

## Data Availability

The data used to support the findings of this study are available from the corresponding author upon reasonable request.
