# Supplementary material for: Dental pain perception and emotional changes: on the relationship between dental anxiety and olfaction
Source: BMC Oral Health. 2023 Mar 26;23:175. doi: 10.1186/s12903-023-02864-9 (PMC10040111; doi:10.1186/s12903-023-02864-9)

1. **General Information**

Patient Name: …………………………………… File Number: …………………………… Age: …………………

Sex: ………………………. Phase: …………………….

1. **Would you please tell us how anxious you feel with your dental visit?**

***Please indicate by inserting "√ " in the appropriate box***

1. *If you went to your dentist for* ***treatment tomorrow****, how would you feel?*

⁮ *Not anxious* ⁮ *Slightly anxious* ⁮ *Fairly anxious* ⁮ *Very anxious* ⁮ *Extremely anxious*

1. *If you were sitting in* ***the waiting room*** *(waiting for treatment), how would you feel?*

⁮ *Not anxious* ⁮ *Slightly anxious* ⁮ *Fairly anxious* ⁮ *Very anxious* ⁮ *Extremely anxious*

1. *If you were about to have* ***a*** ***tooth drilled****, how would you feel?*

⁮ *Not anxious* ⁮ *Slightly anxious* ⁮ *Fairly anxious* ⁮ *Very anxious* ⁮ *Extremely anxious*

1. *If you were about to have your* ***teeth scaled and polished****, how would you feel?*

⁮ *Not anxious* ⁮ *Slightly anxious* ⁮ *Fairly anxious* ⁮ *Very anxious* ⁮ *Extremely anxious*

1. *If you were about to have* ***a local anesthetic injection****, how would you feel?*

⁮ *Not anxious* ⁮ *Slightly anxious* ⁮ *Fairly anxious* ⁮ *Very anxious* ⁮ *Extremely anxious*

1. *If you were about to have* ***a*** ***tooth extraction****, how would you feel?*

⁮ *Not anxious* ⁮ *Slightly anxious* ⁮ *Fairly anxious* ⁮ *Very anxious* ⁮ *Extremely anxious*

1. *If you were about to have* ***a*** ***pulp extirpation****, how would you feel?*

⁮ *Not anxious* ⁮ *Slightly anxious* ⁮ *Fairly anxious* ⁮ *Very anxious* ⁮ *Extremely anxious*

1. *Do you feel upset?*

⁮ *Not at all* ⁮ *Somewhat* ⁮ *Fairly anxious* ⁮ *Moderately so* ⁮ *Very much so*

1. *Do you feel frightened?*

⁮ *Not at all* ⁮ *Somewhat* ⁮ *Fairly anxious* ⁮ *Moderately so* ⁮ *Very much so*

1. *Do you feel nervous?*

⁮ *Not at all* ⁮ *Somewhat* ⁮ *Fairly anxious* ⁮ *Moderately so* ⁮ *Very much so*

1. *Do you feel confused?*

⁮ *Not at all* ⁮ *Somewhat* ⁮ *Fairly anxious* ⁮ *Moderately so* ⁮ *Very much so*

1. *Are you jittery?*

⁮ *Not at all* ⁮ *Somewhat* ⁮ *Fairly anxious* ⁮ *Moderately so* ⁮ *Very much so*

1. *Do you feel that difficulties are piling up so that I cannot overcome them?*

⁮ *Not at all* ⁮ *Somewhat* ⁮ *Fairly anxious* ⁮ *Moderately so* ⁮ *Very much so*

1. *I worry too much over something that really doesn't matter?*

⁮ *Not at all* ⁮ *Somewhat* ⁮ *Fairly anxious* ⁮ *Moderately so* ⁮ *Very much so*

1. *Do you have some unimportant thoughts run through your mind and bothers you?*

⁮ *Not at all* ⁮ *Somewhat* ⁮ *Fairly anxious* ⁮ *Moderately so* ⁮ *Very much so*

1. *Do you* *take disappointments so keenly that you cannot put them out of your mind?*

⁮ *Not at all* ⁮ *Somewhat* ⁮ *Fairly anxious* ⁮ *Moderately so* ⁮ *Very much so*

1. *Do you get in a state of tension or turmoil as you think over your recent concerns and interests?*

⁮ *Not at all* ⁮ *Somewhat* ⁮ *Fairly anxious* ⁮ *Moderately so* ⁮ *Very much so*

1. **Would Please select your pain intensity level by putting “√” in the following scale:**


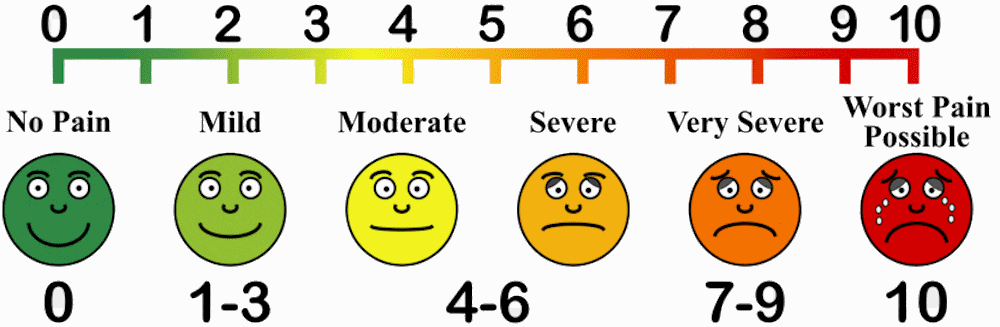

Supplement: Supplementary file 1 — Additional file 1. Questionnaire. [file 12903_2023_2864_MOESM1_ESM.docx]
